# Eating disorders and body image in cystic fibrosis

**DOI:** 10.1016/j.jcte.2021.100280

**Published:** 2021-11-26

**Authors:** Amy Darukhanavala, Lina Merjaneh, Kelly Mason, Trang Le

**Affiliations:** a Division of Pediatric Endocrinology, University of Massachusetts Medical Center, 55 Lake Ave North, Worcester, MA, USA; b Division of Pediatric Endocrinology and Diabetes, Seattle Children’s Hospital, Seattle, WA, USA; c Division of Pediatric Endocrinology and Diabetes, University of Virginia, Charlottesville, VA, USA; d Department of Internal Medicine and Pediatrics, Division of Pediatric Endocrinology, Diabetes, and Metabolism, Virginia Commonwealth University, Richmond, VA, USA

**Keywords:** Cystic fibrosis, Eating disorder, Food, Weight, Anorexia Nervosa, Bulimia nervosa, Binge eating disorder

## Abstract

Eating disorders and disturbed body image have been reported in individuals with cystic fibrosis (CF) and may contribute to poor weight gain, reduced lung function and increased mortality. CF individuals often look and feel different from their peers and bear the additional burden of body-altering side effects of treatment. As a result, the impact of disorders such as binge eating, anorexia nervosa, and bulimia nervosa may adversely affect the social, emotional, and physical development of those with CF.

Multiple risk factors may contribute to the development of an eating disorder in CF. Growth failure is affected by the physical impairments of CF, including pancreatic insufficiency, high energy demands, respiratory infections, and delayed and stunted growth and puberty. Psychological factors, such as CF associated depression and anxiety, intense focus on BMI, lack of control in a chronic disease, and preoccupation with morbidity and mortality, likely further contribute. Exercise inefficiency, secondary to poor lung function, low BMI and pulmonary exacerbations, and the potential for medication manipulation are also additional risk factors.

The intense scrutiny around BMI may lead to a poor relationship with food, including disordered eating habits, abnormal mealtime behaviors, and stressful caregiver-patient interactions regarding meals. This further contributes to a discrepancy between ideal CF nutritional standards and the reality of the challenges of appropriate daily energy intake for an individual with CF.

It is imperative that CF providers are equipped to identify potential eating disorders and disturbed body image in their CF patients. Improved screening and monitoring practices should be developed and implemented, with multidisciplinary support from all CF care team members, including dietitians, mental health professionals, and social workers, to best support holistic care and optimize outcomes. Increased attention to these concerns may help reduce CF related morbidity and mortality.

## Background

Cystic Fibrosis (CF) is a life-threatening disorder with predominantly poor respiratory consequences, however negative endocrine and gastrointestinal outcomes may be severe.1 Maintenance of good nutrition and body mass index (BMI) over the 50th percentile has been shown in multiple studies to improve lung function and reduce mortality risk [2], [3], [4], [5], [6], [7]. The effects of eating disorders, eating disturbances, and body image on nutrition and BMI maintenance is often overlooked in many CF clinics [8]. Unfortunately, the very nature of CF allows for a preoccupation with weight, food, and exercise, resulting in CF itself as a possible risk factor for an eating disorder or poor body image.

Individuals with a chronic and debilitating illness such as CF have the critical challenge of coping with the psychological and physical consequences of the disease. Visible signs of illness, such as poor growth, delayed puberty, malnutrition, and frequent pulmonary exacerbations may cause embarrassment, anxiety, and depression, further contributing to body image disturbances that have harmful consequences, such as eating disorders and disturbed eating. The purpose of this review is to address the prevalence and risk factors for eating disorders/disturbance and disordered eating in the CF population. Additionally, this paper aims to address the need for additional resources for screening, managing and treating these conditions in the CF population.

## Eating disorders and health outcomes

Eating disorders are psychiatric disorders characterized by a persistent disturbance of eating or eating-related behavior that results in the altered consumption or absorption of food and that significantly impairs physical health or psychosocial functioning. The Diagnostic and Statistical Manual 5th edition (DSM-5) include the following classifications: anorexia nervosa, bulimia nervosa, and binge eating disorder [9].

Anorexia nervosa is characterized by restriction of energy intake relative to requirements, leading to a significantly low bodyweight in the context of age, sex, developmental trajectory, and physical health.

It is associated with an intense fear of weight gain or a disturbed body image, or both, which motivates severe dietary restriction or other weight loss behaviors such as purging and excessive exercise. Anorexia nervosa can be qualified as restricting type or binge-eating/purging type based off an assessment of behaviors within three months. Restricting type involves weight loss primarily through dieting, fasting and/or excessive exercise while binge eating or purging type often involves behaviors such as self-induced vomiting, or the misuse of laxatives or other medications. Traditionally, in adults, classification of severity was defined as: mild (BMI > 17), moderate (BMI 16–16.99), severe (BMI 15–15.99) and extreme (BMI < 15). In children and adolescents, a diagnostic criterion was BMI-for-age < 5th percentile. However, the BMI criterion was revised recently to allow for more subjectivity and clinical judgment, allowing professionals to consider an individual's unique growth trajectory and weight history. Anorexia is associated with medical complications due to malnutrition, weight loss and purging, in addition to cognitive and emotional functioning disturbances.

Bulimia nervosa can occur at a normal or elevated weight and is characterized by recurrent episodes of binge eating (at least once per week for 3 months) and compensatory behaviors to prevent weight gain such as self-induced vomiting, inappropriate use of medicines, fasting, or extreme exercise. Binge eating is specifically defined as eating in a discrete period of time an amount of food larger than what most individuals would eat in a similar period of time, as well as a lack of control over the eating during the episodes. Additionally, self-evaluation is unduly influenced by body shape and weight. Disease severity is classified as episodes of inappropriate compensatory behaviors per week with classifications including: mild (1–3 episodes), moderate (4–7 episodes), severe (8–13 episodes), and extreme (14 or more episodes).

In contrast, binge eating disorder is characterized by distressing recurrent episodes of binge eating (at least once a week for three months), as defined above, without regular compensatory behaviors to prevent weight gain. Binge eating episodes are associated with three or more of the following: eating much more rapidly than normal, eating until feeling uncomfortably full, eating large amounts of food when not feeling physically hungry, eating alone because of feeling embarrassed by how much one is eating, and feeling disgusted with oneself, depressed, or very guilty afterwards. There is often marked distress following a binge-eating episode. Disease severity is classified the same way it is for bulimia nervosa [9].

The medical complications of eating disorders are significant and are not limited to those individuals with low BMI. In both the general and CF population, malnutrition from an eating disorder, whether in the presence of low or normal BMI, affects multiple systems. The affects on the endocrine system include growth and pubertal delay, menstrual irregularities, osteoporosis and pathological stress fractures, euthyroid sick syndrome, and hypercortisolism. The cardiovascular system may be affected by mitral valve prolapse, pericardial effusion, arrhythmia or hypotension. The gastrointestinal system may experience gastroparesis or constipation or diarrhea, while complications of the renal system and hematologic system include electrolyte imbalance and anemia, leukopenia or thrombocytopenia, respectively. Finally, the pulmonary system, already significantly affected in the CF population, has an increased risk of pulmonary muscle wasting, decreased pulmonary capacity, respiratory failure, and spontaneous pneumothorax and pneumomediastinum [10].

Eating disorders affect psychological health as well as physical health. Anxiety disorders and depression are among the most common comorbid diagnoses in eating disorders and they are often exacerbated in a state of malnutrition [11]. Studies measuring psychological distress in individuals with CF have found the prevalence of depression is 8–29% in children and adolescents and 13–33% amongst adults, while anxiety in adults ranges from 30 to 33% [12], [13]. However, these conditions reinforce themselves as declining health from an eating disorder results in negative interpersonal experiences that increase the risk for emotional dysfunctional. In other words, while anxiety and depression may contribute to the development of an eating disorder, an eating pathology may aggravate these effective symptoms with greater severity [14].

## Prevalence of eating disorders and body image concerns in CF

The literature provides conflicting studies regarding the prevalence of eating disorders in CF. Additionally, compared to the general population, it remains unclear whether the CF population has a higher, lower or equal prevalence of eating disorders. In a recent meta-analysis conducted by Qian et al that reviewed 33 studies in the general population, the lifetime prevalence of anorexia nervosa, bulimia nervosa and binge eating disorder was 0.16%, 0.63%, and 1.53% respectively. The lifetime prevalence of eating disorders in western countries was 1.89%. The authors found that prevalence studies using DSM-5 criteria were scarce [15].

While several studies report higher prevalence of eating disorders in those with CF [16], [17], [18], other studies report that the prevalence rates of eating disorders do not differ, and may even be lower than, the prevalence rates in the general population [19], [20], [21]. Pumariega et al looked at 108 CF patients between 12 and 21 years of age over the course of three years and identified 13% reached full diagnostic criteria for an eating disorder. They presented with significant weight loss of between 7 and 58% of their total body weight, with an average weight loss of 18%. Many of these individuals also met criteria for major depressive disorder or dysthymic disorder, and when family characteristics of these CF individuals with eating disorders were examined, denial of CF within the family was the most marked family psychological characteristic [18]. Pearson et al reported an incidence of 16.4% of eating disorders in 61 adolescents with CF [17]. Other studies, however, failed to demonstrate an increase in prevalence rates of eating disorders in the CF population. Steiner et al matched CF participants with <75 % ideal body weight with non-CF patients with a known eating disorder and found none of the people with CF met criteria for anorexia nervosa or bulimia nervosa [21]. Raymond et al found eating disorders in two of their control groups but none in the CF sample [19].

It is postulated that CF may even protect against the development of eating disorders due to the high frequency of contact with medical professionals. However, while some studies did not confirm a higher rate of eating disorders in CF, many others demonstrated an elevation in eating disturbances [16], [17], [18]. The true prevalence of eating disorders or eating disturbances in CF is difficult to ascertain due to studies with many methodological limitations, including the use of self-reported measures rather than a diagnostic interview. Additionally, many studies do not further examine symptomology if it does not meet diagnostic criteria. They are therefore unable to provide a formal diagnosis and make it difficult to draw any firm conclusions [21], [17], [18], [19]. Additional studies have paid attention specifically to body image issues in CF. Truby and Paxton noted that the CF population had a lower body mass index than controls and found that those with CF were overall more satisfied with body image than their non-CF counterparts. Those with CF were more likely to perceive their body size as larger than it actually was and had greater satisfaction with their current body size compared to control individuals. Control individuals displayed a high level of body dissatisfaction and desired to be thinner [20].

Studies that did show a higher association of dissatisfaction with body image or an eating disorder with CF revealed differences in gender and disease severity. Tierney et al critically reviewed twelve research studies examining body image in those with CF and found that females had a better body image compared with males due to their preference for a low body weight and thin habitus. Males, however, reported being unhappy with their perceived shape and weight and desired to gain weight and/or become stronger and more muscular despite overestimating their weight [22]. Abbott et al found that females with CF, who tended to be thinner than controls, were happier with their shape. Males with CF, despite overestimating their weight, desired to be heavier more than their non-CF counterparts [23]. Willis et al found that females with CF approve of their slender bodies and even prefer to lose additional weight with 66% following a low calorie diet. On the contrary, males with CF aspired to ideals of masculinity and wanted to gain weight, become more muscular and increase strength. Only 21% followed a low calorie diet [24]. Severity of disease is also thought to contribute to body image [25]. While some individuals with CF appear healthy outwardly, others with more severe disease may have factors which influence body shape, including low body weight, poor posture or barrel chest, and decreased muscle mass. More visibly apparent factors, such as ports and gastrotomy tubes, surgical scars, and supplementary oxygen, may further negatively influence one’s body image.

## Risk factors

The important relationship in CF between nutritional status with pulmonary function and overall survival is well understood [2], [3], [4], [5], [6], [7]. Achieving and maintaining an ideal nutritional status is therefore a critical goal in the treatment of CF, and individuals with CF have a dietary target of between 120% and 150% of the recommended daily allowance with as much as 35–40% of the energy provided by fat. Due to these difficult demands, a frustrating relationship with food and weight may begin to develop, exacerbated by multiple other risk factors for disordered eating and body image disturbances in CF (Table 1). For example, inherent risk factors include weight and growth disturbances, secondary to high energy demands, pancreatic insufficiency, chronic respiratory infections and delayed puberty and growth. Additionally, limited exercise ability, secondary to poor muscle mass and lung function, may further contribute as a risk factor [26]. The preoccupation of CF individuals with food and nutrition presents additional challenges with the burden of eating, including a hyper-focus on weight and BMI, breathlessness when eating, and loss of taste with sinus disease [27]. The ease of weight manipulation through misuse or abuse of pancreatic enzymes, glucocorticoids or insulin (in Cystic Fibrosis Related Diabetes (CFRD)) should not be overlooked. Manipulation of any of these treatments through noncompliance has been identified by some CF individuals as an effective way to lose or maintain weight [18]. Finally, CF, similar to other chronic diseases, is often associated with depression and anxiety, a sense of lack of control, and a preoccupation with morbidity and mortality, increasing the risk for an eating disorder or dissatisfaction with body image [8].

**Table 1:** Risk Factors for Eating Disturbance and Disorders in CF.

• **Weight/Growth Disturbances**
o High energy demands
o Pancreatic insufficiency
o Chronic respiratory infections
o Delayed puberty and growth
• **Exercise Disturbances**
o Poor muscle mass
o Poor lung function
• **Burden of Eating**
o Hyper focus on weight/nutrition/BMI
o Breathlessness when eating
o Loss of taste with sinus problems
• **Ease of Weight Manipulation**
o Pancreatic enzymes
o Glucocorticoids
o Insulin (CFRD)
• **Psychology of Chronic Disease**
o Depression/Anxiety of chronic disease
o Lack of control associated with chronic disease
o Preoccupation with morbidity and mortality

## Disordered eating and abnormal mealtime behaviors

Although the true prevalence of eating disorders in CF is difficult to determine based on existing studies, multiple studies confirmed an increase in eating disturbance in this population [16], [17], [18]. While eating disorders require a formal and clinical diagnosis, disordered eating is defined as irregular behaviors surrounding eating that do not meet criteria for an eating disorder. These include spitting out food, food avoidance, preoccupation with food, bulimic tendencies, and abuse of CF or CFRD related medications [8]. Shearer and Byron evaluated 55 CF individuals ages 11–17 years and found that 24 % showed some evidence of disturbed eating behaviors, the most common was restraint. Of those who either met diagnostic criteria for anorexia or met sub threshold scores for anorexia, 9% were attempting to lose or maintain weight, 4% had fear of gaining weight, 36% felt body shape influenced self-evaluation and 53% felt that their body weight influenced self-evaluation. Another 11%, despite none being overweight, felt fat [16].

The intense focus on nutrition in CF has also been shown to result in worsened mealtime behaviors and distressed parent–child interactions around food. Adherence to dietary recommendations is often poor and results in frequent struggles for the individual with CF and their families. Parents of children with CF tend to view their child’s behavior as especially problematic and more stressful at mealtime in comparison to parents of children without chronic disease. Specifically, they report increased difficulty with poor appetite, problems chewing food, reluctance to eat at designated mealtimes, taking prolonged durations of time to complete a meal, and spitting out food [27], [28], [29]. One study by Sanders et al demonstrated that mealtime behaviors were deemed problematic by 70.4% of parents [30]. Bowen et al showed that parents of children with CF, when compared to controls, gave twice as many commands at mealtime and spoon-fed their children more often. This same study found that children with CF refused food three times more often than control children and were away from the table almost twice as often [31].

## Eating disorders and concerns with body image in the clinical setting

Considering the numerous challenges associated with food, meals and weight, appropriate screening and intervention of CF related eating disorders in the clinical setting is imperative to ensure the success of the CF individual. CF clinics, in addition to pulmonary health care providers, should have access to CF nutritionists, social workers and psychologists. All providers should be allowed opportunities to learn how to accurately assess key markers of negative body image and disordered eating to intervene early, before these negative attitudes and behaviors progress to an eating disorder. Screening practices in the clinic should incorporate an assessment of patient and caregiver stress, anxiety and depression, attitudes towards food and weight, any evidence of food insecurity, non-compliance with medications, dietary assessment, and satisfaction with body image. Unfortunately, there are few reliable and valid eating disorder screeners specifically tailored to those with CF and this significantly limits the ability to accurately assess eating disorders or disturbed eating in this patient population. General eating disorder screening tools have the potential to miscalculate eating disorder risk in those with a chronic illness [8], [32]. For example, some questions may reflect the consequences of CF or its treatment rather than an eating psychopathology, such as a high calorie diet. Finally, non-aversive strategies such as teaching stress management and conflict resolution, designed to promote compliance with dietary recommendations, should be provided to families of those with CF. These interventions should address the parents’ feelings of inadequacy, helplessness and uncertainty about how to cope with their child’s illness, as well as expectations about their child’s dietary intake. Acknowledging the psychological impact of CF, addressing concerns such as anxiety and/or depression in the CF individual, and providing adequate resources for therapy will reduce the likelihood of developing eating disturbance or an eating disorder (Table 2) [8], [33].

**Table 2:** Clinical Needs for Disturbed Eating in CF

**Clinical Needs**	**Suggested Approach**
Limited understanding of the development of an eating disorder	• Conduct focus groups with CF Health Care ProvidersConduct focus groups with CF Patients
Lack of CF-Specific Eating Disorder Screening Tools	• Development of CF specific Screening Tools that address eating disturbance and negative body imageFrequent use of screening tool in CF clinics for consistent assessment and early identification
Limited Training for Health Care providers regarding CF related Eating Disorders	• Provide education to health care providers regarding identifying, addressing and managing eating disordersDevelopment of Resources to provide additional support and education to health care providers
Lack of Eating Disorder Prevention Programs	• Development of a structured prevention program for providers to refer patients of concernCollaboration between CF physician, nutritionist, social worker and psychologist to address patients of concern
Poorly Developed Eating Disorder Intervention Programs	• Development of a structured intervention program that occurs over multiple sessionsTreatment approaches that reduce individual, social-familial, and environmental barriers that interfere with self-management

Under-identification of an eating disorder and missed opportunities for diagnosis and intervention in the CF individual has a range of potential serious consequences. While it is well documented that low BMI and poor nutrition results in decreased lung function and increased mortality in those with CF, additional consequences may include delayed growth and puberty, hypogonadism, menstrual dysfunction, infertility and decreased bone density [1]. Additionally, although associated depression and/or anxiety may already be present, it is crucial to consider the worsening psychological consequences of disturbed eating or an eating disorder [8].

## Knowledge gaps

The stunning medical advances in CF care have fortunately allowed for increased life expectancy, resulting in new concerns with nutrition and body image that were only recently not relevant to the CF population. For example, body image concerns, while once characteristically an issue with being underweight, has more recently been identified with body dissatisfaction and related eating disturbance due to obesity. Studies have shown that with the advent of new CFTR modulators, which not only prolong life but improve weight gain, percent of CF individuals with BMI over 25 has steadily increased. Harindhanavudhi et al. [34] observed a combined overweight and obesity prevalence of 32.2% in their 2020 study, while previous studies revealed an increasing prevalence of 10% (2002) [35], 13% (2004) [36] and 23% (2012) [37]. Additionally, the longer life span results in increased rates of CFRD, requiring insulin, an anabolic hormone that promotes additional weight gain [38].

The literature suggests that individuals with a chronic illness such as CF are at increased risk of adopting disturbed eating or an eating disorder [16], [17], [18]. Patients remain at high risk of jeopardizing their health, and guidelines for screening, identifying and managing these eating disturbances should be established. Additionally, the exact prevalence of eating disturbance and eating disorders in CF remains unknown. Most studies have small sample sizes, inaccurate and non-specific screening tools that are often self-reported, and often fail to identify formal eating disorders vs disturbed eating. Larger studies, with CF specific screening tools, are required. Finally, further evaluation on the effects of CF medical advances on eating disturbance or disorder, as well as body image, is imperative to improved outcomes.

## Conclusion and future directions

It is often assumed that nutritional disorders in CF patients are secondary to the disease process and its effects on the pulmonary, endocrine and gastrointestinal systems. However, findings from the literature suggest the psychological burden of CF may be contributing to poor weight gain, and that eating disorders and disturbed eating must be considered in the differential diagnosis of such problems. The early identification of these disorders in CF patients may contribute significantly to their quality of life and reduce the risk of morbidity and mortality. Currently, there is limited understanding of how eating disorders develop and progress in the CF population and future research should aim to develop resources for CF clinics to identify and manage disturbed eating behaviors. Health care providers can play an important role in regularly monitoring their patients’ psychological status for potential risk factors. The development of adequate and CF-appropriate screening tools, effective eating disorder prevention programs, and interventions aimed at promoting a positive body image should be incorporated into the health care of individuals with CF. Further research is required to better address the risk of eating disorders and disturbed eating in the CF population.

## Funding Sources

This work was supported by the Cystic Fibrosis Foundation EnVision-II CF: Emerging Leaders in CF Endocrinology [grant number DARUKH19GE0 to Dr. Darukhanavala, MERJAN19GE0 to Dr. Meraneh, and MASON19GE0 to Dr. Mason]. The sponsor had no role in study design, in the writing of the report, or in the decision to submit the article for publication.

## CRediT authorship contribution statement

**Amy Darukhanavala:** Conceptualization, Writing – original draft, Writing - review & editing, Visualization. **Lina Merjaneh:** Writing – original draft, Writing - review & editing. **Kelly Mason:** Writing – original draft, Writing - review & editing. **Trang Le:** Conceptualization, Writing - review & editing, Supervision.

## Declaration of Competing Interest

The authors declare that they have no known competing financial interests or personal relationships that could have appeared to influence the work reported in this paper.
